# Sleeping

**Published:** 1882-09

**Authors:** 


### SLEEPING.

**It is nothing short of murderous for one person to sleep habitually in a room less than twelve feet each way; and even then the fire-place should be kept open,** and a door ajar, or the windows raised at bottom, or lowered at top, (both better;) this creates a draught up the chimney, and carries off much of the foul air generated during sleep. A little fire, or a lamp, or jet of gas burning in the fire-place, increases the draught. As the air we breathe is the chief agent for removing all impurities from the blood, the more effectual as it is purer, it must be plain to all that the room in which we spend a clear third of our entire existence should contain the purest air possible, and that this must have an immense influence on the health. Hence, our chambers should be large and airy—the higher above the ground the better—with windows facing the south, so as to have all the benefit of sunlight and warmth, to keep them dry and cheerful. Besides a few handsome pictures or paintings on the walls, illustrating what is beautiful and elevating, there should be no furniture except a table, a dressing-bureau, and a few chairs, all without covering. With the exception of the bedding and a clean dry towel, there should be no woven fabric, neither carpet, curtains, nor hanging garments; for these, especially if woolen, retain odors, dust, dampness, and seeds of corruption and disease for months. There should be a hearth-rug at the bedside to prevent the bare feet from coming in contact with the cold floor, on getting out of a warm bed. No liquid except a pitcher of cold water should be allowed to remain five minutes in a sleeping-room. The deadly carbonic acid gas which comes from the lungs at every out-breathing of the sleeper, rises to the ceiling in warm weather, but falls to the floor when the room is freezing cold. Hence, in summer, the purest and coolest air in a room is near the floor; in winter, the foulest.

To Sleep Soundly.—Inability to sleep, as a growing habit, is the first step toward certain madness; in every disease it is an omen of ill. Hence, to cultivate sound sleep, do not sleep a moment in the day-time; go to bed at a regular hour, and never take a "second nap" after waking of yourself in the morning. Take nothing after dinner but a piece of cold bread and butter, and one cup of hot drink · *not* China tea, as it makes many wakeful. Never go to bed cold or very hungry, nor with cold feet. Read nothing after supper, listen to nothing, talk about nothing of a very exciting character; avoid carefully every domestic unpleasantness, as to child, servant, husband, or wife. Let no angry word be spoken or thought harbored for a single instant after tea-time, for death may come before the morning-light. Grown persons generally require seven hours' sleep in summer, and eight in winter. Few indeed, except invalids, will fail to sleep well who go to bed at a regular early hour, on a light supper, in a large room, and clean, comfortable bed, if there is no sleeping in the day-time, and not more than seven hours in any twenty-four are passed in bed. One week's faithful trial will prove this. Children, and all persons at school or engaged in hard study, should take all the sleep they can get, and should never be waked up in the morning after having gone to bed at a regular early hour. Every humane parent will make it a religious duty to arrange that every child shall go to bed in an affectionate, loving, and glad spirit. If wakeful during the night, get up, draw on the stockings, throw back the bed-cover to air it, walk the floor in your night-gown, with the mouth closed, all the while rubbing the skin briskly with both hands, until cooled off and a little tired. Except from August first to October first, in fever and ague localities, a chamber window should be open two or three inches at least.—See Dr. Hall on "Sleep."

